# Supplementary material for: Collaborative intelligence in AI: Evaluating the performance of a council of AIs on the USMLE
Source: PLOS Digit Health. 2025 Oct 9;4(10):e0000787. doi: 10.1371/journal.pdig.0000787 (PMC12510544; doi:10.1371/journal.pdig.0000787)
Supplement: S2 File — (ZIP) [file pdig.0000787.s002.zip › S2 Data Raw data files containing unprocessed question inputs and LLM outputs including transcripts of deliberations/STEP3_Question-114-1_Type-MC-NJ.docx]

**Facilitator** **[Round 1]:** A 74-year-old man who had a stroke 2 years ago is admitted to the hospital from the nursing care facility because of a 4-day history of reduced urinary output, and worsening abdominal pain, nausea, and lethargy. The stroke had resulted in right-sided hemiparesis and difficulties with speech and swallowing. Medical history is also significant for hypertension. The patient has been hospitalized twice during the past 3 months for pneumonias that did not respond to oral antibiotic therapy in the nursing facility. He underwent bladder catheterization during each of the recent hospitalizations for a total of 6 days to monitor urine output. The catheter was removed prior to each discharge. Bladder catheterization attempts during the past several days in response to his current symptoms have been unsuccessful. Now on admission, the patient appears agitated and confused. Vital signs are temperature 36.9°C (98.4°F), pulse 110/min, respirations 24/min, and blood pressure 160/100 mm Hg. Physical examination discloses jugular venous distention, 8 cm at 45 degrees. Lungs are clear to auscultation. Auscultation of the heart discloses regular tachycardia with no gallops. There is fullness and tenderness to palpation of the low central abdomen but no rebound or guarding. Examination of the extremities shows 1+ pedal edema bilaterally. The prostate is normal in size and has no palpable nodules or tenderness. Results of laboratory studies are shown: Serum Urea nitrogen - 73 mg/dL Creatinine - 4.2 mg/dL Calcium - 8.6 mg/dL Na⁺ - 134 mEq/L K⁺ - 5.6 mEq/L Cl⁻ - 100 mEq/L HCO3⁻ - 20 mEq/L Mg2⁺ - 2.0 mEq/L Glucose - 126 mg/dL Phosphorus - 4.8 mEq/L Blood Hematocrit - 32% Hemoglobin - 10.6 g/dL WBC - 8200/mm³ Platelet count - 359,000/mm³ Ultrasonography shows distention of the bladder and bilateral hydronephrosis. ECG shows sinus tachycardia. Which of the following is the most appropriate next step? (A) Administration of bethanechol (B) Administration of prazosin (C) Hemodialysis (D) Placement of a suprapubic catheter (E) Ultrafiltration

**AI_E:** RESTATING THE QUESTION: We are reviewing the case of a 74-year-old man, a victim of previous stroke, suffering from reduced urinary output, abdominal pain, nausea, and lethargy. He's also agitated and confused. He has a history of pneumonia and hypertension, along with unsuccessful bladder catheterization attempts over recent days. Laboratory studies and a physical examination have been provided, along with an ultrasound report indicating a distended bladder and bilateral hydronephrosis, and ECG indicating sinus tachycardia. The question is asking what the next appropriate action should be from the options given. PLANNING: We must consider the patient's symptoms, his lab results, age, and medical history. His lab test shows signs of impaired kidney function (increased urea and creatinine). Also, the ultrasound results indicating bladder distention and bilateral hydronephrosis suggest possible urinary obstruction, which can increase pressure on the kidneys, further impairing their function. CLINICAL REASONING: Being an elderly patient with a history of stroke, he could have developed neurogenic bladder, where control over bladder function is lost due to brain or nerve problems. This condition, combined with an unsuccessful catheterization procedure, could have led to bladder distention and consequently bilateral hydronephrosis, an indication of urinary obstruction. Additionally, his increased agitation and confusion could be linked to uremic encephalopathy due to the impaired kidney function. CONCLUDING: Considering the clinical symptoms, lab results, and ultrasound findings, it's clear the patient is suffering from acute kidney injury (AKI) likely due to postrenal obstruction. Treatment for postrenal AKI requires relieving the obstruction. Therefore, the most appropriate next step would be to effectively drain his bladder. RESPONSE: The most appropriate next step in treating this patient is (D) Placement of a suprapubic catheter. The suprapubic catheter will help to relieve the urinary obstruction, thereby alleviating pressure on the kidneys, reducing his symptoms, and allowing the reintroduction of normal bladder function. Bethanechol and prazosin are medicines used to treat bladder problems, but they are not the optimal treatments for a physically blocked pathway. Hemodialysis and ultrafiltration are important treatments for severe kidney dysfunction, but may not be necessary once the cause of the renal failure, in this case the urinary obstruction, has been addressed.

**AI_A:** 1) RESTATING THE QUESTION: The clinical scenario presents a 74-year-old man with a medical history of stroke leading to right-sided hemiparesis and speech and swallowing difficulties, and hypertension. Recently, his past three months were marked by recurrent pneumonia that required hospitalization and catheterization each time. Currently, he experiences reduced urination and worsening abdominal symptoms, which have prompted his latest admission. On examination, he is found to be agitated and confused, and shows signs of high blood pressure, tachycardia, and jugged venous distention. His abdomen is tender to palpation and his extremities show minor edema. He has abnormal urea nitrogen and creatinine levels, indicating a possible issue with kidney function. Ultrasound results suggest bladder distention and bilateral hydronephrosis. The question seeks to determine the most appropriate next step in the treatment of the patient. 2) PLANNING: The assumptions we can make are that his current symptomatology and clinical findings are possibly due to a urological problem especially considering the ultrasound findings. We should question whether this could be a result of his previous catheterizations or complications from his stroke which triggered neurogenic bladder dysfunction. Given the evidence, prioritizing the assumption that he has an obstruction that has led to urinary retention would lead us in the most clinical significant direction. 3) CLINICAL REASONING: We begin by noting his high urea nitrogen and creatinine levels in addition to his elevated blood pressure and tachycardia, these are signs of renal impairment, possibly due to an obstructive uropathy. Furthermore, the distention of his bladder and bilateral hydronephrosis confirm this. Urinary retention might be a complication from his previous catheterizations or could stem directly from his stroke. Neurogenic bladder dysfunction, which can be a consequence of cerebrovascular accident, could lead to impairment of bladder control. 4) CONCLUDING: To decompress his distended bladder and relive the obstruction, we need a more invasive intervention, since previous attempts at catheterization have failed. 5) RESPONSE: Thus, considering the patient's condition and ultrasound findings, the most appropriate next step would be the placement of a suprapubic catheter (D). This procedure will allow direct drainage of the urinary bladder through an incision in the lower abdomen, thereby bypassing potential obstructions in the urethra. It should help to alleviate his symptoms and improve his renal function by relieving the urinary retention.

**AI_D:** (1) RESTATING THE QUESTION: This question focuses on a 74-year-old male patient who presents with a collection of symptoms including reduced urinary output, abdominal pain, nausea, lethargy, agitation, and confusion. He also has a history of stroke, hypertension, recurrent pneumonia, and has previously undergone bladder catheterization. His laboratory studies reveal elevated urea nitrogen and creatinine levels amongst others, hinting at a potential renal issue, while ultrasonography confirms bladder distention and bilateral hydronephrosis. We're asked to determine the most appropriate next step in managing his condition. (2) PLANNING: Because of his history of bladder catheterization, stroke and current symptoms of reduced urine output and distended bladder, one assumption is that he might be suffering from an obstructive urinary pathology, such as urinary retention. This would be in line with the elevated urea nitrogen and creatinine levels, which indicate impaired kidney function potentially due to back-flow of urine. However, this does not explain his other symptoms like agitation and confusion, which might be due to uremic encephalopathy caused by the accumulation of toxins usually expelled in urine, or other causes. Therefore, an alternate assumption might be that he has acute kidney injury secondary to post-renal obstruction. To evaluate this, we would need to consider the signs of urinary obstruction and kidney dysfunction, such as anuria, distended bladder, and elevated renal parameters. (3) CLINICAL REASONING: Urinary obstruction can lead to hydronephrosis, which is distention of the renal calyces and pelvis due to urine accumulation, and it can in turn cause kidney damage if prolonged and severe. In line with this thought, the patient's previous bladder catheterizations might have caused damage leading to obstruction and resultant hydronephrosis, as identified by the ultrasonography report. The confused state of the patient and his elevated serum urea nitrogen and creatinine levels suggest acute kidney injury (AKI), possibly due to the hydronephrosis. This indicates that the problem might be post-renal AKI, which is caused by some form of obstruction to the urinary tract. (4) CONCLUDING: Thus, the main issue seems to be a urinary tract obstruction leading to post-renal AKI. The most important step in managing this would be to relieve the obstruction so as to restore urine flow and possibly reverse the AKI. (5) RESPONSE: Therefore, the most appropriate next step would be (D) Placement of a suprapubic catheter. Suprapubic catheterization is a good option because it can help bypass potential obstructions in the urethra or bladder neck, as could be the case here given the patient's history and current symptoms. This would thus effectively alleviate the urinary retention and assisting in resolving the post-renal AKI. The other options are less appropriate as they either target hypertension (prazosin - B), stimulate bladder contraction (bethanechol - A) or treat severe fluid and electrolyte imbalances or remove waste products and toxins when the kidney function is significantly reduced (hemodialysis - C and ultrafiltration - E), but without directly addressing the actual cause of this patient's symptoms, the obstruction.

**AI_C:** RESTATING THE QUESTION: What is the best course of action for our patient, a 74-year-old man with a history of a stroke, hypertension, multiple hospitalizations for pneumonia, and recent difficulties with bladder catheterization. His main symptoms include reduced urinary output, abdominal discomfort, distention of the bladder, bilateral hydronephrosis, and altered mental status. His lab results also indicate elevated urea nitrogen and creatinine levels, suggestive of possible kidney stress or damage. We are asked to consider whether we should administer bethanechol, prazosin, conduct hemodialysis, place a suprapubic catheter or perform ultrafiltration. PLANNING: Bethanechol is a parasympathomimetic that acts on the muscarinic acetylcholine receptors present in the urinary bladder. Prazosin is an alpha-1 antagonist, primarily used to treat high blood pressure. Hemodialysis is a treatment for kidney failure, usually invoked when kidney function has significantly deteriorated. Suprapubic catheterization involves surgically placing a catheter into the bladder via the lower abdomen, bypassing the urethra. Ultrafiltration is a process that removes excess fluid from the blood, typically in heart failure patients. Given the patient's history and symptoms, it's most plausible that he's experiencing acute urinary retention, which has led to hydronephrosis and kidney failure. The most direct approach would be to relieve this obstruction. CLINICAL REASONING: The patient's history suggests a correlation between his stroke and subsequent neurological symptoms (hemiparesis, speech difficulty, altered mental status) with the bladder dysfunction, as the bladder's smooth muscle contraction/relaxation coordination is controlled by neural inputs (parasympathetic and sympathetic responses). Utilizing drugs like bethanechol or prazosin might not be effective or lead to side effects as a drug-induced hormonal regulation might be insufficient or flawed in a “neurogenic bladder” situation. Hemodialysis or ultrafiltration are more appropriate for addressing acute kidney injury or chronic kidney disease, and heart failure respectively, but do not fundamentally address this patient's issue which seems to originate from obstruction and distention of the urinary pathways. Removing the obstruction, which seems to be the source of the problem, is likely the best course of action. CONCLUDING: Jugular venous distention and pedal edema suggest volume overload, which, in this patient’s case, most likely originated from an inability to adequately excrete urine due to a urinary obstruction. Elevated urea and creatinine are signs of kidney injury, possibly caused by acute urinary retention leading to acute kidney injury. Failed catheterizations may be caused by urethral blockage or damage. Given these considerations, the best course of action would be to immediately relieve the urinary tract obstruction. RESPONSE: Given the patient's symptoms and the ineffectiveness of previous catheterization attempts, the placement of a suprapubic catheter (D) appears to be the most appropriate action. Placement of this catheter, which avoids the urethra, will immediately relieve thehydro-nephrosis and urinary obstruction, and potentially reduce the stress on the kidneys, allowing for kidney function to stabilize or improve.

**AI_B:** 1) RESTATING THE QUESTION: The clinical question asks about the most appropriate next step in management for our elderly patient who had a past medical history of stroke resulting in right-sided hemiparesis, issues with speech and swallowing, hospitalized multiple times for poorly managed pneumonia, and hypertension. The patient is now presented with reduced urinary output since less than a week, abdominal pain, lethargy, nausea, elevated blood pressure, regular tachycardia, distended jugular veins, and bilateral pedal edema. Laboratory tests results, ultrasonography, and ECG findings suggest renal impairment with bladder distention and bilateral hydronephrosis. 2) PLANNING: The assumptions we can make from the case presented are that the patient is experiencing urinary retention due to his medical history of stroke, which could impact bladder function, and his recent symptoms of reduced urine output and fullness of low central abdomen. One alternative assumption could be that the urinary retention is due to other factors, like prostate enlargement, which is quite common in older males, but physical examination found the prostate to be normal. Another alternative assumption is that the previous catheterizations have caused some form of injury or scar tissue formation preventing further successful catheterizations. However, given the immediate history of neurogenic dysfunction secondary to stroke, it's clinically prioritized to assume that leftover effects from the stroke might be contributing to the urinary retention as the bladder can be affected by neurogenic dysfunction, leading to its inability to empty properly. 3) CLINICAL REASONING: Stroke can cause neurogenic bladder, which can manifest as an overactive or underactive bladder. Overactive bladder would manifest with symptoms of urgency, frequency, and urge incontinence. Conversely, underactive bladder or acontractile bladder (also known as detrusor underactivity) would present with symptoms of urinary retention, which is what our patient is presenting with. The bilateral hydronephrosis noted in the imaging can be secondary to the chronic urinary retention caused by the underactive bladder. The raised urea and creatinine levels are a direct result of the hydronephrosis, indicating a backflow effect of urine causing damage to the kidneys, which is termed obstructive uropathy. 4) CONCLUDING: Given the clinical context of the patient's presenting symptoms, the development of an acontractile bladder due to his stroke history, unsuccessful attempts of bladder catheterization, and the severeness indicated by bilateral hydronephrosis, it is vital to relieve the urinary retention as soon as possible to avoid further obstruction, renal damage or potential progression to renal failure. 5) RESPONSE: Therefore, I would recommend the Placement of a Suprapubic Catheter (option D) as the most appropriate next-step management in this scenario. This procedure would provide relief of the urinary retention by providing a new tract for urine to be expelled, thus mitigating the risk of further hydronephrosis or renal failure. The other options such as administration of bethanechol or prazosin (A & B) might help promote urinary function in certain situations, but in this acute case of severe urinary retention with kidney damage, they might not be as effective or timely as the placement of a suprapubic catheter. Hemodialysis (option C) could be considered later if renal function continues to worsen despite resolving the urinary retention. And, ultrafiltration (option E) while useful in managing fluid overload states, wouldn't provide a direct solution to the urinary retention. Therefore, the best next step in management at this point would be the placement of a suprapubic catheter (option D).

**Facilitator** The consensus among the AI responses is that the most appropriate next step in managing the 74-year-old patient's condition is the placement of a suprapubic catheter (D). The patient's symptoms, medical history, and current clinical findings suggest he is suffering from acute urinary retention, likely due to an obstructive uropathy. This obstruction has led to bladder distention and bilateral hydronephrosis, as confirmed by ultrasound, and is likely causing his elevated urea nitrogen and creatinine levels, indicative of renal impairment. The patient's history of stroke could have resulted in neurogenic bladder dysfunction, contributing to his urinary retention. Previous attempts at bladder catheterization have been unsuccessful, suggesting possible urethral blockage or damage. Therefore, the placement of a suprapubic catheter, which bypasses the urethra, is recommended to relieve the urinary obstruction, alleviate the patient's symptoms, and potentially improve his renal function. Other options such as the administration of bethanechol or prazosin, hemodialysis, or ultrafiltration, while potentially useful in certain situations, do not directly address the patient's immediate issue of urinary obstruction and are therefore considered less appropriate in this specific clinical scenario.
